# Colourimetric LAMP detection of *Escherichia coli* in bovine milk using a simplified DNA preparation method

**DOI:** 10.1007/s11259-026-11576-8

**Published:** 2026-10-14

**Authors:** Tewodros Fentahun Jember, Mark Edward Westman, Sameer Dinkar Pant, Seyed Ali Ghorashi

**Affiliations:** 1 Faculty of Science and Health, School of Agricultural, Environmental and Veterinary Sciences, Charles Sturt University, Wagga Wagga, Australia; 2 College of Veterinary Medicine and Animal Sciences, University of Gondar, Gondar, Ethiopia; 3 Elizabeth Macarthur Agricultural Institute (EMAI), Department of Primary Industries and Regional Development (DPIRD), Menangle, NSW 2658 Australia; 4 Gulbali Institute, Charles Sturt University, Boorooma Street, Wagga Wagga, NSW 2678 Australia

**Keywords:** Dairy cattle, *Escherichia coli*, Mastitis, DNA preparation, Colourimetric LAMP, Veterinary science

## Abstract

*Escherichia coli* (*E. coli*) is a common cause of bovine mastitis, and rapid detection from milk is important for timely disease management. However, molecular assays such as colourimetric loop-mediated isothermal amplification (LAMP) are often limited by laboratory-based DNA extraction. This study evaluated a simplified HotSHOT (HS) DNA preparation method for extracting *E. coli* DNA from bovine milk and assessed its compatibility with colourimetric LAMP and conventional PCR. Pasteurised bovine milk spiked with *E. coli* was used for analytical evaluation, and culture‑positive clinical milk samples were tested to assess diagnostic performance. The HS method was compared with five commercial DNA extraction kits and a direct boiling method without centrifugation. Performance was assessed using limit of detection (LOD) expressed as colony-forming units per millilitre. Using the HS method, the LOD was 7.9 × 10⁴ CFU/mL for LAMP and 7.9 × 10³ CFU/mL for PCR. PCR performance of the HS method was comparable to the QIAamp and Norgen commercial kits, while LAMP sensitivity was ten- to a hundred-fold lower than the best kit-based methods. Direct boiling showed markedly reduced sensitivity, with an LOD of 7.9 × 10⁸ CFU/mL. Among 17 culture-confirmed clinical mastitis milk samples positive for *E. coli*, LAMP detected 15 (88.2%) and PCR detected all 17 (100%). These results show that simplified DNA preparation can support molecular detection of *E. coli* in bovine milk and provide a practical basis for future development of field-deployable diagnostics in resource-constrained settings.

**Supplementary Information:**

The online version contains supplementary material available at https://doi.org/10.1007/s11259-026-11576-8.

## Introduction

Bovine mastitis remains a major health challenge in dairy production and leads to reduced milk yield, compromised animal welfare, and increased antimicrobial use (Ghumman et al. 2026, Ramuada et al. 2024; Hogeveen et al. 2011, Tomanic et al. 2025). Bacterial culture is considered the reference method for identifying mastitis-causing pathogens, but it is time-consuming and usually requires milk samples to be transported to diagnostic laboratories (Servellon et al. 2025; Ramuada et al. 2024). As a result, treatment decisions are often delayed or made before pathogen identification is available (Wilm et al. 2021; Preine et al. 2022).

Several rapid cow-side tests, such as the California Mastitis Test and somatic cell count-based methods, are widely used to detect mastitis (Peckler and Adcock 2025; Ferronatto et al. 2018). While these tests are effective for identifying inflammation of the mammary gland, they do not identify the causative bacterial agent. This lack of pathogen-specific information often leads to non-specific treatment, which may reduce treatment success and contribute to unnecessary antimicrobial use (Saeed et al. 2024; Ajose et al. 2022).

Molecular diagnostic methods offer sensitive and specific detection of mastitis causing bacteria directly from milk samples (Duarte et al. 2015; Kajdanek et al. 2024; Koskinen et al. 2009). Conventional polymerase chain reaction (PCR) has been widely adopted for this purpose, but it requires thermal cyclers, stable electricity, and trained personnel (Shome et al. 2011; Syring et al. 2012; Hiitio et al. 2015; Riffon et al. 2001). These requirements limit its use outside laboratory environments and restrict its application as a routine on-farm diagnostic tool (Wong et al. 2020; Poussard et al. 2025; Ghorashi et al. 2022; Garner et al. 2023).

Loop-mediated isothermal amplification (LAMP) has emerged as a practical alternative to PCR for decentralised testing (Notomi et al. 2000). LAMP operates under isothermal conditions and does not require complex instrumentation (Cui et al. 2024; Garner et al. 2023; Notomi et al. 2000). The development of colourimetric LAMP assays has further simplified detection by allowing results to be interpreted visually through colour change, instead of relying on turbidity measurement, fluorescence detection, or gel electrophoresis, which require additional laboratory equipment (Tanner et al. 2015). These features make LAMP suitable for rapid testing in non-laboratory settings and as a potential cow side molecular assay (Kapalamula et al. 2021; Pascual-Garrigos et al. 2021).

Among mastitis-causing pathogens, *Escherichia coli* (*E. coli*) is one of the most common environmental bacteria associated with both clinical and subclinical mastitis in dairy cattle (Goulart and Mellata 2022). Infection often arises from environmental contamination such as bedding, manure, and suboptimal housing conditions, and outbreaks are frequently linked to wet and unsanitary farm environments (Goulart and Mellata 2022). Rapid identification of *E. coli* is important because infections can progress quickly and may require management strategies that differ from those used for contagious mastitis pathogens (Goulart and Mellata 2022).

Despite the advantages of molecular assays, their performance strongly depends on the quality of the extracted DNA (Ravine and Suthers 2012; Ghorashi et al. 2013). DNA extraction is often the most critical and limiting step in molecular diagnostics (Paul et al. 2020). Rapid DNA preparation methods are typically crude and may contain PCR inhibitors that reduce assay sensitivity (Date Chong et al. 2023). In contrast, commercial DNA extraction kits generally provide higher quality DNA but require laboratory equipment, multiple reagents, and higher costs (Silva et al. 2023). This mismatch between simple amplification methods (e.g. LAMP) and complex DNA extraction processes (e.g. column-based commercial kits) limits the broader adoption of molecular diagnostics outside laboratory settings (Zou et al. 2017).

The aim of this study was to develop and evaluate a colourimetric LAMP assay for the detection of *E. coli* in bovine milk and to assess its compatibility with a simplified HotSHOT (HS) DNA preparation method. Both analytically controlled spiked milk samples and culture-confirmed clinical milk samples were used to assess assay performance. The performance of the HS method was compared with five commercial DNA extraction kits and a direct boiling method. Extracted DNA was tested using the developed LAMP assay and conventional PCR, and assay sensitivity and specificity were evaluated using the limit of detection expressed as colony-forming units per millilitre (CFU/mL).

## Materials and methods

### Preparation of *E. coli*–spiked milk samples

**Fig. 1:**
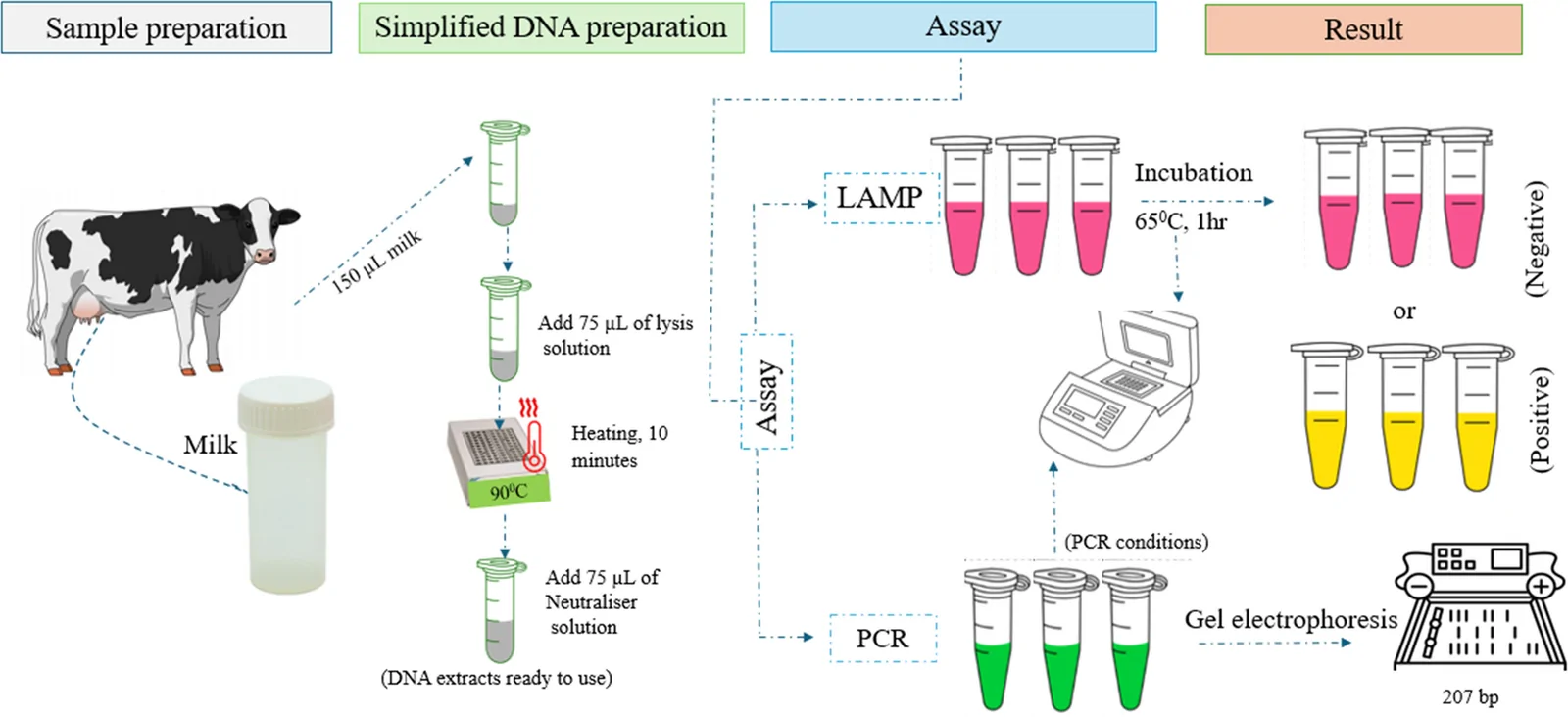
Overview of the LAMP based workflow for detection of *E. coli* in bovine milk. Milk was sampled from the affected quarter and processed by a simplified HS DNA preparation. The prepared DNA was tested by colourimetric LAMP targeting the *uidA* gene, with results read visually as a colour change from pink to yellow. Conventional PCR, yielding a 207 bp product, was performed in parallel as a reference method for comparison

An overview of the complete workflow, from milk sampling through HS DNA preparation to colourimetric LAMP and PCR detection, is shown in Fig. 1. A commercial pasteurised full-cream bovine milk was used to prepare *E. coli*–spiked samples for assay evaluation. *E. coli* colonies were obtained from the Veterinary Diagnostic Laboratory at Charles Sturt University and were originally isolated from bovine mastitis milk samples and identified as *E. coli* using selective differential media and standard biochemical tests. A loopful of colonies was inoculated into Luria-Bertani (LB) broth and cultured overnight at 37 °C with shaking. The identity of the isolate was confirmed by sequencing (NCBI accessions PV472575 to PV472578), and a characterised reference strain will be included in future work.

Following incubation of the LB broth, 1 mL of the overnight culture was added to 9 mL of pasteurised full-cream milk in a sterile Falcon tube to prepare the initial dilution. A tenfold serial dilution series was then prepared from this suspension to generate dilutions ranging from 10⁻¹ to 10⁻¹⁰. All dilutions were prepared and tested in triplicate.

LB agar was prepared using tryptone, yeast extract, and sodium chloride as previously described (MacWilliams and Liao 2006). The medium was autoclaved at 121 °C for 30 min, poured into Petri dishes, and allowed to solidify. From each dilution, 100 µL was spread onto LB agar plates. The agar plates were incubated at 37°C for 18 h for colony counting.

After incubation, colonies were counted on plates containing between 30 and 300 colonies. Colony-forming units per mL were calculated based on the number of colonies observed, the volume plated, and the corresponding dilution factor. Each dilution was plated and counted directly, so the reported CFU per mL reflected the viable count in each tested tube rather than a value back calculated from the starting culture. DNA extracted from each serial dilution using the seven DNA extraction methods was used as a template for colourimetric LAMP and PCR assays. Results were expressed as limit of detection (LOD) to enable relative comparison of the seven DNA extraction methods. The LOD was defined as the lowest bacterial concentration, in CFU per mL, at which a positive result was obtained in at least two of three replicates for a given assay and extraction method.

### DNA extraction

The optimised HS DNA preparation method consists of a lysis solution containing 0.2 mM disodium EDTA and 25 mM NaOH adjusted to pH 12.0, and a neutralisation solution containing 40 mM Tris HCl adjusted to pH 1.9. For each extraction, 150 µL of milk was mixed with 75 µL of lysis solution in a 1.5 mL tube, homogenised by pipetting and gentle finger flicking, and heated at 90 °C for 10 min on a heat block. After heating, 75 µL of neutralisation solution was added and mixed, giving a final volume of 300 µL. No centrifugation was required. DNA was extracted from each serial dilution of *E. coli*–spiked milk, ranging from 10⁻¹ to 10⁻¹⁰, using the optimised HS method and six additional DNA extraction methods listed in Table 1. Commercial kits were used according to the manufacturers’ instructions. For the direct boiling method, 150 µL of milk was heated at 100 °C for 10 min on a heat block, and the upper liquid phase was taken directly as template without centrifugation. Direct boiling without centrifugation was included because it is the simplest heat‑based extraction that could be performed on farm with only a heat block, matching the equipment requirements of the HS method. It therefore served as a heat‑only comparator for the alkaline lysis and neutralisation chemistry of HS. This procedure differs from conventional laboratory boiling protocols, in which bacterial cells are pelleted by centrifugation and resuspended in buffer before boiling. All extractions were performed using samples contained in sterile Falcon tubes.

**Table 1:** DNA extraction methods evaluated in this study

Method ID	Extraction method	Source	Extraction mechanism
1	Wizard^®^ Genomic DNA Purification Kit (Gram Positive and Negative Bacteria) (Promega Corporation, Australia)	Commercial kit	Enzymatic, Mechanical
2	ReliaPrep™ Blood gDNA Miniprep System (Promega Corporation, Australia)	Commercial kit	Enzymatic, Mechanical
3	QIAmp DNA Microbiome Kit (QIAGEN, USA)	Commercial kit	Mechanical, Enzymatic, Chemical
4	Milk Bacterial DNA Isolation Kit (Norgen Biotek Corporation, Ontario, Canada)	Commercial kit	Mechanical, Enzymatic
5	Quick-DNA Fecal/Soil Microbe DNA Miniprep Kit (Zymo Research Corporation, Australia)	Commercial kit	Mechanical, Enzymatic
6	Direct boiling method (no centrifugation)	Non-commercial	Thermal
7	Optimised HS method	Non-commercial (test)	Chemical, Thermal

Pasteurised full cream milk spiked with distilled water instead of overnight *E. coli* culture was used as a negative extraction control. The detailed extraction procedure for the optimised HS method has been described previously (Jember et al. 2026a).

### LAMP and PCR primer design

Colourimetric LAMP primers were designed to detect *E. coli* by targeting the *uidA* gene, which encodes beta-glucuronidase. The target gene sequence was obtained from the NCBI GenBank database under accession number KT311783.1. The *uid*A gene, which encodes beta-glucuronidase, was selected because it is a widely accepted molecular marker specific to *E. coli*. Multiple candidate LAMP primer sets were generated in PrimerExplorer V5 (http://primerexplorer.jp/e/index.html) provided by Eiken Chemical Co. in Tokyo, Japan, and the final set was selected on the basis of predicted melting temperature, GC content, absence of strong primer dimers and hairpins, and location within a conserved region of the gene. The LAMP primer set consisted of an outer forward primer (EC-F3), an outer backward primer (EC-B3), a forward inner primer (EC-FIP), a backward inner primer (EC-BIP), a forward loop primer (EC-LF), and a backward loop primer (EC-LB).

Primer binding sites were mapped relative to the *uidA* gene sequence of *E. coli* (GenBank accession KT311783.1). Nucleotide positions were determined for the outer and loop primers, while the inner primers FIP and BIP were designed as composite primers derived from internal regions of the target gene, consistent with standard LAMP architecture.

**Fig. 2:**
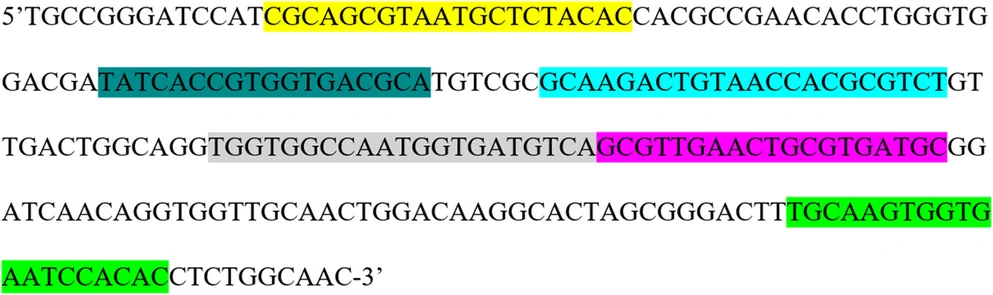
Schematic representation of LAMP primer binding sites within the *uidA* gene of *E. coli* (GenBank accession KT311783.1). Each primer is colour coded to indicate its specific binding region. The locations of outer primers (F3 and B3) and loop primers (LF and LB) are shown with nucleotide coordinates relative to the reference sequence. Inner primers FIP and BIP are composite primers comprising two target specific regions and are therefore not represented by single continuous binding positions

The EC F3 and EC B3 primers were also used as forward and reverse primers for conventional PCR amplification, generating an expected amplicon size of 207 base pairs. All primers were synthesised by Sigma-Aldrich Pty Ltd in Australia. Primer sequences are listed in Table 2, and primer binding locations within the target sequence are illustrated in Fig. 2. Primer sequences are colour coded in Table 2 to allow clear visual correspondence with their binding locations shown in Fig. 2.

**Fig. 3:**
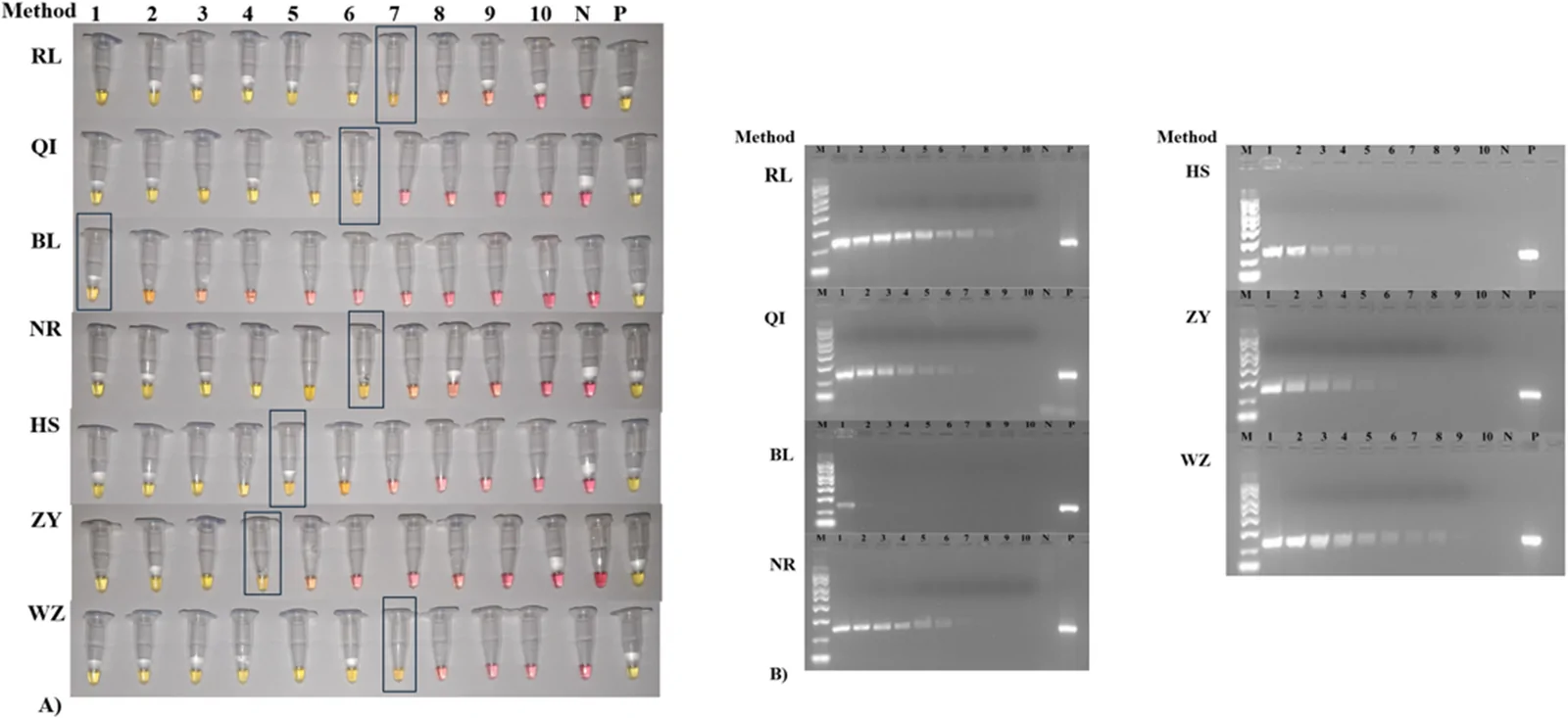
Determination of LOD for LAMP and PCR using *E. coli* spiked milk samples. (**A**) Colourimetric LAMP results obtained from tenfold serial dilutions of full cream milk spiked with *E. coli*. Labels 1 to 10 correspond to dilutions 10⁻¹ to 10⁻¹⁰. Each dilution was tested in triplicate. A colour change from pink to yellow was interpreted as a positive result. Boxed tubes indicate the dilution at which the LOD was determined for each DNA extraction method. N indicates negative control and P indicates positive control. (**B**) Agarose gel electrophoresis of PCR amplicons generated using DNA extracted from the same serially diluted milk samples. Lanes 1 to 10 correspond to dilutions 10⁻¹ to 10⁻¹⁰. M indicates DNA ladder, N negative control, and P positive control. PCR products were separated on 1.5% agarose gels stained with GelRed. LOD values for each DNA extraction method are expressed as CFU/mL. The boxed tube in (**A**) and the last visible band in (**B**) indicate the limit of detection for each method; values are given in Table 3. DNA extraction methods are abbreviated as follows: RL, ReliaPrep Blood gDNA Miniprep System; QI, QIAamp DNA Microbiome Kit; BL, direct boiling method; NR, Milk Bacterial DNA Isolation Kit; HS, optimised HS method; ZY, Quick DNA Fecal Soil Microbe DNA Miniprep Kit; WZ, Wizard Genomic DNA Purification Kit

**Table 2:** Sequences and positions of primers targeting the *uidA* gene of *E. coli* used for LAMP and PCR

Primer name	Primer Sequence (5’−3’)	Position of primers in uidA gene^a^
EC-F3	CGCAGCGTAATGCTCTACAC	198–217
EC-B3	GTGTGGATTCACCACTTGCA	385–404
EC-FIP	AGACGCGTGGTTACAGTCTTGC-GAACACCTGGGTGGACGA	F2: 224–241 and F1c: 266–287
EC-BIP	TGGTGGCCAATGGTGATGTCA-TCCCGCTAGTGCCTTGTC	B1c: 301–321 and B2: 364–381
EC-LF	TGCGTCACCACGGTGATA	242–259
EC-LB	GCGTTGAACTGCGTGATGC	322–340

^a^ Positions are given relative to the *uidA* gene sequence of *E. coli* (GenBank accession KT311783.1)

### Colourimetric LAMP assay

The colourimetric LAMP assay was initially developed and optimised using purified *E. coli* genomic DNA extracted from the same bacterial isolate described above using the Wizard SV Genomic DNA Purification System (Promega). The assay was performed using a modified WarmStart^®^ Colourimetric LAMP 2× Master Mix (New England Biolabs, USA). Each reaction was carried out in a final volume of 20 µL containing 10 µL of 2× LAMP master mix, primers at final concentrations of 0.2 µM each for EC-F3 and EC-B3, 0.4 µM each for EC-LF and EC-LB, and 1.6 µM each for EC-FIP and EC-BIP, 5 µL of nuclease-free water, and 3 µL of extracted DNA. The LAMP and PCR reactions used 3 µL and 2 µL of template in total volumes of 20 µL and 25 µL respectively, so the proportion of template was similar, and the same extracted DNA was used for both assays.

For negative controls, both nuclease free water and DNA extracted from non-spiked pasteurised milk were used. For positive controls, 2 µL of *E. coli* genomic DNA at 1 ng/µL, extracted using the same commercial kit, was included.

LAMP reactions were incubated at 65 °C on a heat block for 60 min. The relatively long incubation reflects amplification from a crude milk extract, and the reduced speed advantage of LAMP under these conditions is addressed in the discussion. For DNA extracted using the optimised HS method, the incubation time was extended by an additional 30min. This adjustment was made to allow sufficient amplification from a crude DNA preparation that may contain lower DNA concentrations or residual inhibitors compared to purified kit-based extractions. This extended incubation time was applied consistently for all experiments using HS-extracted DNA. According to the manufacturer’s instructions, a colour change from pink to yellow was interpreted as a positive result, while no colour change was considered negative.

### Conventional PCR assay

Conventional PCR was performed using a Rotor Gene™ 6000 thermal cycler (Qiagen, Melbourne, Australia). Each reaction was carried out in a final volume of 25 µL containing 5.0 µL of 5× GoTaq Green Flexi Reaction Buffer, 1.5 µL of MgCl₂ (25 mM), 2.0 µL of dNTP mix (1.25 mM each), 0.3 µL of Taq DNA polymerase (5 U/µL; Promega, Australia), 1.0 µL each of EC-F3 and EC-B3 primers (2 µM), and 2.0 µL of extracted DNA. Nuclease free water was added to reach the final reaction volume. Negative and positive controls were prepared as described for the colourimetric LAMP assay.

PCR amplification was performed with an initial denaturation at 95 °C for 5 min, followed by 30 cycles of denaturation at 95 °C for 1 min, annealing at 56 °C for 45 s, and extension at 72 °C for 70 s, with a final extension at 72 °C for 8 min. The PCR products (10 µL) were analysed by agarose gel electrophoresis on a 1.5% agarose gel and visualised using a Gel Doc XR+ imaging system (Gel Doc XR+, Bio-Rad, USA).

### Limit of detection (analytical sensitivity)

The performance of different DNA extraction methods was compared based on the limit of detection (LOD) for both colourimetric LAMP and PCR assays. For each serial dilution, bacterial concentration was determined by colony counting and expressed as CFU/mL. The LOD was defined as the lowest bacterial concentration at which a positive result was obtained in at least two of three replicates for a given assay and DNA extraction method. Where minor variation in LOD was observed across the three technical replicates for a given extraction method, the value obtained in the majority of the replicates was taken as the representative LOD (Supplementary Fig. 1).

### Diagnostic sensitivity assessment using clinical milk samples

Following the comparative evaluation of DNA extraction methods using spiked milk samples, the diagnostic sensitivity of the optimised HS method was assessed using clinical milk samples obtained from the Elizabeth Macarthur Agricultural Institute in Australia. A total of 17 clinical bovine milk samples that were culture positive for *E. coli* were included. Identification of *E. coli* was confirmed by matrix-assisted laser desorption ionisation time-of-flight mass spectrometry. DNA was extracted from these samples using the optimised HS method and tested using colourimetric LAMP and conventional PCR assays. Diagnostic sensitivity and specificity were calculated using a 2× 2 contingency table, with bacterial culture as the reference method. Calculations were performed using the MedCalc online diagnostic test evaluation tool (https://www.medcalc.org/calc/diagnostic_test.php).

### Specificity assessment

The specificity of the colourimetric LAMP and PCR assays were evaluated by testing the primers against a panel of non-target bacterial species selected to assess potential cross reactivity. The panel included *Streptococcus uberis*,* Pasteurella multocida*,* Mycoplasma gallisepticum*,* Erysipelothrix* species, *Staphylococcus aureus*,* Mycoplasma synoviae*,* Streptococcus zooepidemicus*, multidrug resistant *Staphylococcus aureus*,* Pseudomonas aeruginosa*,* Enterococcus faecalis*, and Salmonella Typhimurium. Genomic DNA from *E. coli* was used as a positive control, while distilled water was included as a negative control.

In addition, the specificity of the outer primers EC‑F3 and EC‑B3 was assessed in silico by BLASTn against the NCBI core nucleotide database (accessed September 2026), with the maximum number of target sequences set to 500. Searches were performed without restriction and then restricted in turn to *Shigella*, *Klebsiella pneumoniae*, *Enterobacter cloacae*, *Citrobacter* and *Serratia marcescens*. A full match was defined as 100% identity across the whole primer length.

### Statistical analysis

The representative limit of detection for each method and assay was taken as the value obtained in the majority of the three replicates. No formal statistical test of reproducibility was applied, because the replicates were technical rather than independent.

## Results

### Development and optimisation of colourimetric LAMP and PCR assays

Under the optimised conditions, the colourimetric LAMP assay produced a clear colour change from pink to yellow in the presence of *E. coli* DNA, while no colour change was observed in negative control reactions containing nuclease-free water.

Similarly, conventional PCR amplification using EC-F3 and EC-B3 primers consistently generated the expected 207 bp amplicon from *E. coli* genomic DNA, with no amplification detected in negative control reactions. These results confirmed that both assays were functional and suitable for subsequent analytical evaluation.

### Comparison of DNA extraction methods and assay limits of detection using *E. coli* spiked pasteurised bovine milk

The limits of detection agreed to within one dilution across the three technical replicates for each extraction method and assay (Table 3; Supplementary Fig. 1). Using the optimised HS method, colourimetric LAMP detected *E. coli* at concentrations of approximately 7.9 × 10⁴ CFU/mL, while PCR detected *E. coli* at a representative LOD of approximately 7.9 × 10³ CFU/mL. The QIAamp DNA Microbiome Kit and the Milk Bacterial DNA Isolation Kit gave PCR limits of detection comparable to HS (7.9 × 10³ CFU/mL), but tenfold lower LAMP limits (7.9 × 10³ CFU/mL).

**Table 3:** LOD of *E. coli* determined by LAMP and PCR across three replicate samples using different DNA extraction methods

DNA extraction method	LAMP LOD (CFU/mL)			PCR LOD (CFU/mL)		
	E1	E2	E3	E1	E2	E3
RL	7.9 × 10²	7.9 × 10³	7.9 × 10²	7.9 × 10¹	7.9 × 10²	7.9 × 10¹
QI	7.9 × 10³	7.9 × 10³	7.9 × 10³	7.9 × 10³	7.9 × 10³	7.9 × 10²
NR	7.9 × 10³	7.9 × 10³	7.9 × 10³	7.9 × 10³	7.9 × 10³	7.9 × 10³
HS	7.9 × 10⁴	7.9 × 10⁴	7.9 × 10⁴	7.9 × 10³	7.9 × 10⁴	7.9 × 10³
ZY	7.9 × 10⁵	7.9 × 10⁵	7.9 × 10⁴	7.9 × 10³	7.9 × 10⁴	7.9 × 10⁴
WZ	7.9 × 10²	7.9 × 10²	7.9 × 10²	7.9 × 10¹	7.9 × 10¹	7.9 × 10²
BL	7.9 × 10⁸	7.9 × 10⁸	7.9 × 10⁷	7.9 × 10⁸	7.9 × 10⁷	7.9 × 10⁸

Values represent limits of detection expressed as CFU/mL. E1, E2, and E3 indicate three replicate samples tested from a single DNA extraction. RL: ReliaPrep Blood gDNA Miniprep System (Promega Corporation, Australia); NR: Milk Bacterial DNA Isolation Kit (Norgen Biotek Corporation, Canada); ZY: Quick DNA Fecal Soil Microbe DNA Miniprep Kit (Zymo Research Corporation, Australia); QI: QIAamp DNA Microbiome Kit (QIAGEN, USA); HS: optimised HotSHOT method; WZ: Wizard Genomic DNA Purification Kit (Promega Corporation, Australia); BL: direct boiling method. The limit of detection agreed across the three replicates to within one dilution, and the representative value was that obtained in the majority of the three replicates

The Quick DNA Fecal/Soil Microbe DNA Miniprep Kit showed higher detection limits, with LOD values of approximately 7.9 × 10⁵ CFU/mL for colourimetric LAMP and 7.9 × 10⁴ CFU/mL for PCR. The ReliaPrep Blood gDNA Miniprep System and the Wizard Genomic DNA Purification Kit demonstrated the highest sensitivity among the commercial kits, with limits of detection of 7.9 × 10² CFU/mL for LAMP and 7.9 × 10¹ CFU/mL for PCR. In contrast, the direct boiling method gave an LOD of 7.9 × 10⁸ CFU/mL for both assays, several orders of magnitude higher than that of the HS and kit-based methods. A detailed summary of the limits of detection obtained for each DNA extraction method using colourimetric LAMP and PCR is presented in Table 3, and a graphical comparison of detection limits is shown in Fig. 3.

### Clinical validation and diagnostic sensitivity of the colourimetric LAMP and PCR assays

The diagnostic sensitivity of the colourimetric LAMP and PCR assays was assessed using 17 clinical bovine milk samples that were culture positive for *E. coli*. DNA was extracted from all samples using the optimised HS method and tested by colourimetric LAMP and conventional PCR.

Among the 17 clinical milk samples tested, 15 were positive by colourimetric LAMP, corresponding to a diagnostic sensitivity of 88.24% (95% CI: 0.63–0.98). All 17 samples were positive by PCR, indicating a diagnostic sensitivity of 100%. No false positive reactions were observed in negative control samples for either assay.

**Fig. 4:**
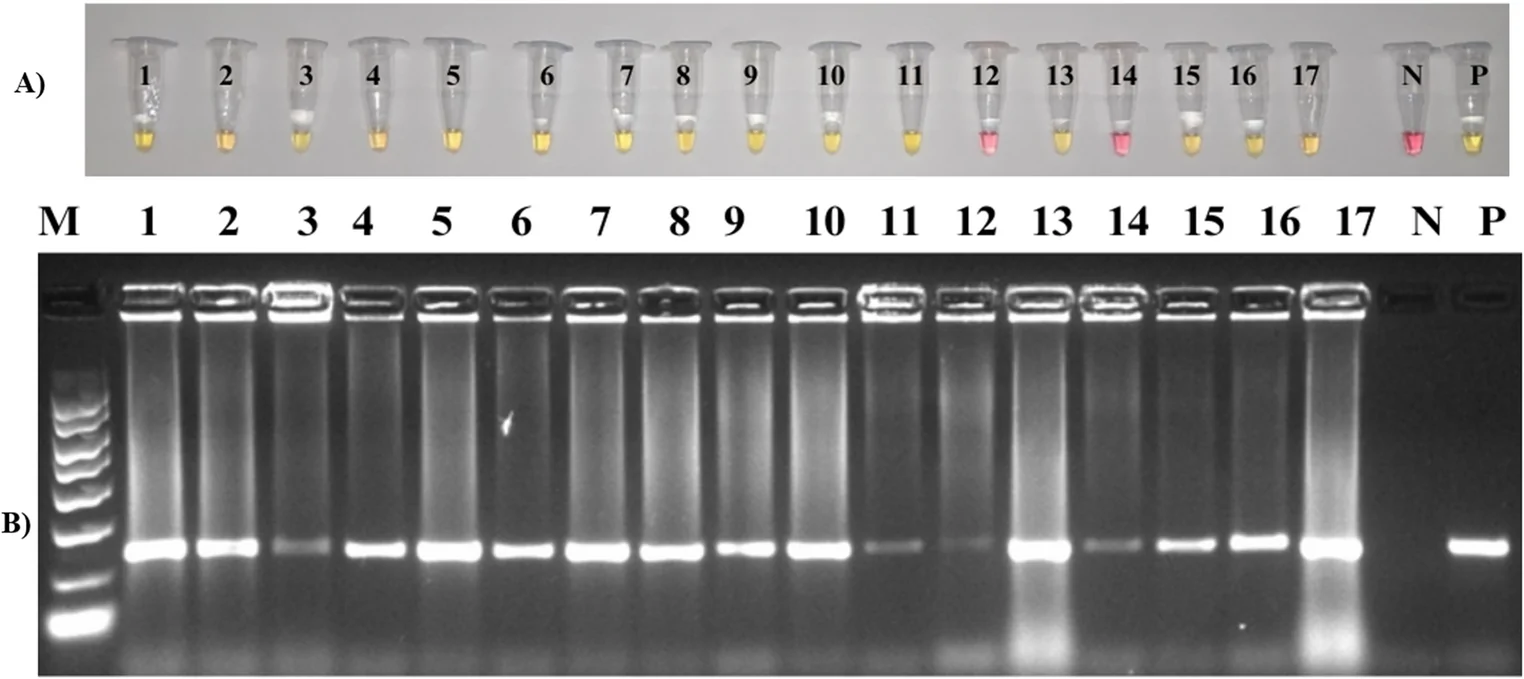
Clinical validation of the LAMP and PCR assays for detection of *E. coli* in bovine milk samples. (**A**) Colourimetric LAMP results for 17 clinical milk samples (tubes 1–17) using template DNA extracted with the optimised HS method. N indicates negative control and P indicates positive control. (**B**) Agarose gel electrophoresis of PCR amplicons generated from the same clinical milk samples (*n* = 17) targeting the *uidA* gene of *E. coli*. Template DNA was extracted using the optimised HS method. PCR products were separated on 1.5% agarose gel stained with GelRed and visualised under UV illumination. M indicates DNA ladder, N negative control, and P positive control

Representative colourimetric LAMP results and corresponding PCR amplification profiles for the clinical milk samples are shown in Fig. 4.

### Specificity of the colourimetric LAMP and PCR assays

**Fig. 5:**
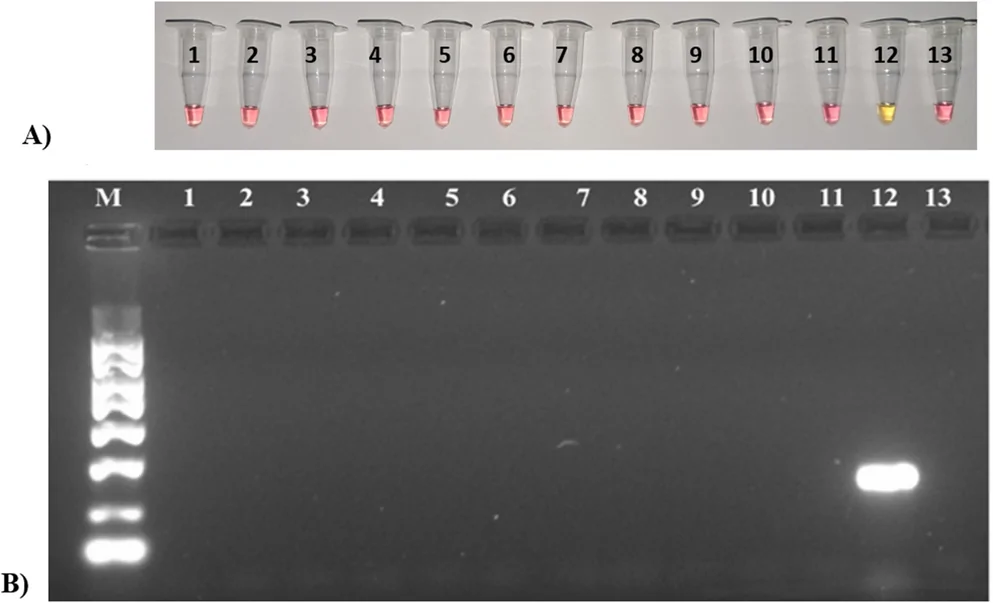
Specificity assessment of the colourimetric LAMP and PCR assays. PCR products were analysed on a 1.5% agarose gel. The expected amplicon size was 207 bp (**A**) Colourimetric LAMP results and (**B**) PCR amplification using primers designed for *E. coli*, M indicates the DNA ladder, lanes 1–11 correspond to non-target bacterial isolates, lane 12 corresponds to *E. coli* and lane 13 represents the negative control with no template DNA

The colourimetric LAMP and PCR assays showed no cross-reactivity with any of the 11 non-target bacterial species tested (Table 4; Fig. 5). Only *E. coli* produced a positive result in both assays. Sequencing of selected PCR amplicons confirmed their identity as *E. coli*, and the sequences were deposited in NCBI under accession numbers PV472575, PV472576, PV472577, and PV472578.

**Table 4:** Analytical specificity of the LAMP and PCR assays against non-target bacterial species

Bacterial species tested	LAMP result	PCR result
*Streptococcus uberis*	-	-
*Pasteurella multocida*	-	-
*Mycoplasma gallisepticum*	-	-
*Erysipelothrix* spp.	-	-
*Staphylococcus aureus*	-	-
*Mycoplasma synoviae*	-	-
*Streptococcus zooepidemicus*	-	-
Methicillin-resistant *Staphylococcus aureus*	-	-
*Pseudomonas aeruginosa*	-	-
*Enterococcus faecalis*	-	-
Salmonella Typhimurium	-	-
*Escherichia coli*	+	+
Negative control	-	-

**“+”** = positive; **“-”** = negative, Two isolates of *S. aureus* were tested: one methicillin-sensitive and one methicillin-resistant

In silico analysis showed that both outer primers matched *E. coli* sequences with 100% identity and full query coverage. Full matches were also found for *Shigella* species. No full-length match was found for *K. pneumoniae*, *E. cloacae*, *Citrobacter* species or *S. marcescens*, for which only partial alignments were obtained.

## Discussion

The optimised HS method produced DNA of sufficient quality for both LAMP and PCR detection of *E. coli* in bovine milk, with LOD comparable to several commercial extraction kits. These results demonstrate that a colour-based LAMP assay combined with a simplified DNA preparation method can provide a practical approach for detecting this pathogen. This supports the concept that complex and resource-intensive DNA extraction procedures are not always necessary for reliable molecular detection, particularly for decentralised or near-farm testing applications (Shwani et al. 2023). The LAMP chemistry, the *uid*A target, and the alkaline lysis approach are each established and are not presented as novel in themselves. The contribution of this work is the integration of a crude DNA preparation performed directly on milk with a visual colourimetric readout, benchmarked against five commercial kits.

A key outcome of this work was the consistent performance of the HS method, with LOD values agreeing to within one dilution across technical replicates. Repeatable performance is a critical requirement for on-farm diagnostics, where testing conditions may vary (da Silva et al. 2021). In contrast, direct boiling of whole milk without centrifugation gave an LOD of 7.9 × 10⁸ CFU/mL. This method was included as the simplest field-compatible alternative to HS, requiring the same equipment and no centrifugation. Its poor performance indicates that heat alone does not release amplifiable DNA from milk, and that the alkaline lysis and neutralisation steps account for the performance of HS. The high LOD is likely to reflect carryover of milk fat, proteins and divalent cations that inhibit amplification (Habib et al. 2024; Chen et al. 2019), rather than an inherent limitation of boiling. Conventional boiling protocols that pellet and wash bacterial cells would be expected to perform considerably better (Cui et al. 2024), but they add a centrifugation step that the HS method avoids.

The LAMP assay developed in this study demonstrated analytical sensitivity within the same order of magnitude as PCR when tested using *E. coli* spiked pasteurised bovine milk samples and DNA extracted using the optimised HS method (LAMP: 7.9 × 10⁴ CFU/mL; PCR: 7.9 × 10³ CFU/mL). Although PCR consistently achieved lower detection limits, both assays detected *E. coli* within a similar range, with PCR reaching the 10³ CFU/mL range and LAMP the 10⁴ CFU/mL range, indicating that the HS method provided DNA of sufficient quality for reliable detection by either method. This finding aligns with previous studies reporting that LAMP is a robust amplification method capable of tolerating certain inhibitors and operating effectively under isothermal conditions (Jackson et al. 2020). However, the results also confirm that LAMP performance remains dependent on DNA quality, reinforcing that DNA preparation is still a critical step even for isothermal amplification assays (Boza et al. 2024; Nwe et al. 2024; Moehling et al. 2021). The LAMP incubation in this study was 60min, and together with the 30-minute HS DNA preparation, the total time to a result was about 90 min, which is longer than the fastest reported LAMP run times and reduces its usual speed advantage. This reflects amplification from a crude milk extract, and optimising the enzyme, master mix, and reaction conditions is a clear priority for shortening the assay in future work.

The assay did not reach the analytical sensitivity typically reported for real‑time PCR, which was not evaluated directly in this study, and its total time to result was similar to a laboratory qPCR run. Its intended value therefore lies not in replacing qPCR but in where it can be performed. Point‑of‑care diagnostics are generally judged on whether they are fit for purpose in their intended setting, accepting some loss of analytical sensitivity in exchange for affordability, simplicity and independence from laboratory equipment (Land et al. 2019). Real‑time PCR requires an optical thermal cycler and trained personnel to interpret amplification curves, and in routine mastitis diagnostics milk samples are sent to a diagnostic laboratory, with results typically available on the same or the following day (Hiitiö et al. 2015). Portable real‑time PCR instruments are available, but they remain considerably more expensive than the heat block required for the HS and LAMP workflow. In contrast, the HS method costs approximately AU$0.50 per sample, compared with AU$6 to AU$22 for commercial extraction kits (Jember et al. 2026b), and together with colourimetric LAMP gives a visual colour change that can be read without specialised training, although borderline colour changes may still require confirmation. Because the LOD obtained here lies below bacterial counts associated with clinical infection (Haider et al. 2023), the assay is best suited to the rapid identification of *E. coli* in clinical mastitis. However, LAMP did not detect 2 of 17 culture‑positive clinical samples, and laboratory qPCR remains the more appropriate choice for low bacterial loads and subclinical infection.

*E. coli* was selected as the target organism in this study due to its relevance as a common environmental cause of bovine mastitis and its association with rapid disease progression, making it a suitable model pathogen for evaluating field-deployable molecular diagnostics. The bacterial load in milk, expressed as CFU/mL, is an important indicator of udder infection and mastitis status. In this study, the LOD of the LAMP and PCR assays using DNA extracted by the optimised HS method were 7.9 × 10^4^ and 7.9 × 10³ CFU/mL, respectively. These detection limits are below the threshold associated with active mastitis, as bacterial counts above 1 × 10⁵ CFU/mL have been reported as indicative of infection in dairy cattle (Haider et al. 2023). Although lower LOD values for LAMP-based detection of *E. coli* have been reported elsewhere, such as the 5.7 × 10² CFU/mL reported by Cui et al. (2024), those studies relied on laboratory-adapted boiling protocols involving bacterial culture and multiple centrifugation steps. In contrast, DNA extraction in the present study was performed directly from milk, a complex biological matrix containing fat, proteins, and other components known to interfere with molecular amplification. Under these conditions, the HS method represents a compromise between analytical sensitivity and operational simplicity, supporting its use in resource constrained laboratory settings and near farm applications.

When applied to clinical milk samples, the LAMP assay detected *E. coli* in 15 of 17 culture-positive samples, with a diagnostic sensitivity of 88.24%, whereas PCR detected *E. coli* in all 17 samples tested. The reduced sensitivity observed for LAMP in clinical samples may be attributed to lower bacterial loads, heterogeneous distribution of bacteria in milk, or the presence of inhibitory components that affect colour development or amplification efficiency (Nwe et al. 2024). These factors are well recognised challenges in direct milk testing and highlight the trade-off between assay simplicity and analytical sensitivity in field-deployable diagnostics (Kase and Pfefer 2016, Nwe et al. 2024).

High specificity was observed for both LAMP and PCR, with no cross-reactivity detected against non-target bacterial species. This indicates that primers targeting the *uidA* gene provide specific detection of *E. coli*, which is essential for mastitis diagnostics where milk samples may contain a diverse microbial population. In silico analysis supported this, since the primers did not fully match the closely related coliform mastitis pathogens *Klebsiella*,* Enterobacter*,* Citrobacter* or *Serratia.* They did match *Shigella*, which is genetically almost indistinguishable from *E. coli* and is not a cause of bovine mastitis, so this has no practical consequence for the intended use of the assay. Accurate pathogen identification is critical for guiding appropriate treatment and avoiding unnecessary antimicrobial use (Yamin et al. 2023; Bhattacharya 2013; Ku et al. 2023).

Overall, this study demonstrates that combining a colour-based LAMP assay with the HS DNA preparation method provides a balanced solution between analytical performance and operational simplicity. While PCR remains more sensitive, the HS-LAMP workflow reduces reliance on laboratory infrastructure, specialised equipment, and trained personnel, supporting its potential application as a near-farm or on-farm diagnostic tool (Bath et al. 2020; Lai et al. 2023; Lee et al. 2019; Yang et al. 2022; Sowmya et al. 2012).

Despite the promising results obtained in this study, several limitations should be acknowledged. The number of clinical milk samples used for assay validation was relatively small, which limits the precision of the estimated diagnostic sensitivity and may not fully capture the variability present under field conditions. In addition, clinical samples were pre-characterised as *E. coli* positive by bacterial culture, and inclusion of a larger and more diverse sample set, including culture-negative samples and samples containing mixed infections, would allow more comprehensive evaluation of diagnostic specificity. The assay was evaluated using laboratory-prepared spiked milk samples and DNA extracted under controlled conditions, and performance may vary when applied directly on farm, where environmental factors, sample handling, and operator variability could influence results. In addition, the boiling comparator did not include the centrifugation and resuspension steps used in conventional boiling protocols, so its results should not be taken as representative of boiling extraction in general. Furthermore, although the colour-based readout simplifies result interpretation, borderline colour changes may introduce subjectivity, particularly at low bacterial concentrations. We also did not assess the purity of the HS extracts spectrophotometrically, since crude alkaline preparations are not well suited to standard purity ratios; the preparation was instead judged functionally by its ability to support reliable amplification. Future work should focus on larger-scale clinical validation and evaluation under true field conditions to further assess assay robustness, usability, and impact on mastitis management practices (Jones et al. 2019).

## Supplementary Information

Below is the link to the electronic supplementary material.

Supplementary Material 1 (PNG 139 KB)

## Data Availability

1. Sequence data is available in NCBI database under accession numbers: PV472575, PV472576, PV472577, and PV472578.2. Colorimetric LAMP data and serial dilution data are already included in the manuscript.

## Declarations

### Generative AI and AI-assisted technologies

This manuscript was prepared by the authors. Generative AI and AI-assisted tools were used only for language editing and to improve grammar and clarity of the text. No AI tools were used to generate scientific content, analyse data, interpret results, or draw conclusions. All ideas, analyses, and interpretations are the original work of the authors, who take full responsibility for the content of the manuscript.

## References

[CR1] Ajose DJ, Oluwarinde BO, Abolarinwa TO, Fri J, Montso KP, Fayemi OE, Aremu AO, Ateba CN (2022) Combating bovine mastitis in the dairy sector in an era of antimicrobial resistance: ethno-veterinary medicinal option as a viable alternative approach. Front Vet Sci 9:800322 10.3389/fvets.2022.800322 PMC9014217 35445101

[CR2] Bath C, Scott M, Sharma PM, Gurung RB, Phuentshok Y, Pefanis S, Colling A, Singanallur Balasubramanian N, Firestone SM, Ungvanijban S, Ratthanophart J, Allen J, Rawlin G, Fegan M, Rodoni B (2020) Further development of a reverse-transcription loop-mediated isothermal amplification (RT-LAMP) assay for the detection of foot-and-mouth disease virus and validation in the field with use of an internal positive control. Transbound Emerg Dis 67:2494–2506 10.1111/tbed.13589 32311239

[CR3] Bhattacharya S (2013) Early diagnosis of resistant pathogens: how can it improve antimicrobial treatment? Virulence 4:172–184 10.4161/viru.23326 PMC3654618 23302786

[CR4] Boza JM, Manning JC, Erickson DC (2024) Comparison and Optimization of Simple DNA Extraction Methods for LAMP-Based Point-of-Care Applications Employing Submillimeter Skin Biopsies. ACS Omega 9:38855–38863 10.1021/acsomega.4c05025 PMC11411550 39310140

[CR5] Chen YX, Bi CB, Tong SM, Gong Z, Hou HS (2019) An improved and reliable method for microalgae direct PCR. J Appl Phycol 31:2411–2421

[CR6] Cui S, Wei Y, Li C, Zhang J, Zhao Y, Peng X, Sun F (2024) Visual loop-mediated isothermal amplification (LAMP) assay for rapid on-site detection of *Escherichia coli* O157: H7 in milk products. Foods 13:2143 10.3390/foods13132143 PMC11241362 38998648

[CR7] da Silva RP, Assaf AV, Pereira AC (2021) Reproducibility and validity of diagnostic tests. In: Peres MA, Antunes JLF, Watt RG (eds) Oral Epidemiology (Springer International Publishing: Cham)

[CR8] Date Chong M, Sheehan S, Battaglia J, Wescott DJ, Wallin J (2023) Comparative study of Rapid DNA versus conventional methods on compromised bones. Forensic Sci Int Genet 63:102825 10.1016/j.fsigen.2022.102825 36592573

[CR9] Duarte CM, Freitas PP, Bexiga R (2015) Technological advances in bovine mastitis diagnosis: an overview. J Vet Diagn Invest 27:665–672 10.1177/1040638715603087 26450837

[CR11] Jember FT, Westman ME, Pant SD, and Ghorashi SA (2026a) Field-deployable molecular workflow for detection, resistance screening, and genotyping of Staphylococcus aureus in bovine mastitis. Vet J 316:106583 10.1016/j.tvjl.2026.106583 41654106

[CR12] Jember FT, Westman ME, Pant SD, Ghorashi SA (2026b) Comparative evaluation of DNA extraction methods for detecting mastitis-associated bacteria in milk. Vet J 319:106823 10.1016/j.tvjl.2026.106823 42586307

[CR13] Ferronatto JA, Ferronatto TC, Schneider M, Pessoa LF, Blagitz MG, Heinemann MB, Della AMMP, Libera, Souza FN (2018) Diagnosing mastitis in early lactation: use of Somaticell^®^, California mastitis test and somatic cell count. Italian J Anim Sci 17:723–729

[CR14] Garner C, Stephen C, Pant SD, Ghorashi SA (2023) Comparison of PCR-HRM, colorimetric LAMP and culture based diagnostic assays in the detection of endometritis caused by Streptococcus equi subsp. zooepidemicus in mares. Vet Res Commun 47:495–509 10.1007/s11259-022-10047-0 PMC9765344 36538151

[CR15] Ghorashi SA, Bradbury JM, Ferguson-Noel NM, Noormohammadi AH (2013) Comparison of multiple genes and 16S-23S rRNA intergenic space region for their capacity in high resolution melt curve analysis to differentiate *Mycoplasma gallisepticum* vaccine strain ts-11 from field strains. Vet Microbiol 167:440–447 10.1016/j.vetmic.2013.09.032 24238667

[CR16] Ghorashi MS, Pant SD, Ghorashi SA (2022) Comparison of colourimetric loop-mediated isothermal amplification (LAMP), PCR and high-resolution melt curve analysis and culture-based diagnostic assays in the detection of three *Salmonella* serotypes in poultry. Avian Pathol 51:476–487 10.1080/03079457.2022.2101916 35833568

[CR17] Goulart DB, Mellata M (2022) *Escherichia coli* Mastitis in Dairy Cattle: Etiology, Diagnosis, and Treatment Challenges. Front Microbiol 13:928346 10.3389/fmicb.2022.928346 PMC9301288 35875575

[CR18] Habib S, Azmai MNA, Yasin IM, Masdor NA, Said NAM, Yasid NA (2024) Streamlined boiling lysis DNA extraction for Gram-positive aquaculture pathogen *Streptococcus agalactiae*. Arch Microbiol 206:435 10.1007/s00203-024-04163-5 39417886

[CR19] Haider Ali M, Ikram I, Shahzadi, Muhammad Asif Raza (2023) Polymeric Nanoparticles for Bovine Mastitis Treatment. Springer

[CR20] Hiitio H, Riva R, Autio T, Pohjanvirta T, Holopainen J, Pyorala S, Pelkonen S (2015) Performance of a real-time PCR assay in routine bovine mastitis diagnostics compared with in-depth conventional culture. J Dairy Res 82:200–208 10.1017/S0022029915000084 25704849

[CR22] Hogeveen Henk S, Pyorala K, Persson Waller, Joe S, Hogan TJGM, Lam SP, Oliver YH, Schukken HW, Barkema, Eric Hillerton J (2011) Current status and future challenges in mastitis research. In Proceedings of the 50th annual meeting of the National Mastitis Council, 23–26

[CR23] Jackson KR, Layne T, Dent DA, Tsuei A, Li J, Haverstick DM, Landers JP (2020) A novel loop-mediated isothermal amplification method for identification of four body fluids with smartphone detection. Forensic Sci Int Genet 45:102195 10.1016/j.fsigen.2019.102195 31835180

[CR24] Jones G, Bork O, Ferguson SA, Bates A (2019) Comparison of an on-farm point-of-care diagnostic with conventional culture in analysing bovine mastitis samples. J Dairy Res 86:222–225 10.1017/S0022029919000177 31038086

[CR25] Kajdanek A, Kluska M, Matusiak R, Kazimierczak J, Dastych J (2024) A rapid and inexpensive PCR test for mastitis diagnosis based on NGS data. Pathogens 13:423 10.3390/pathogens13050423 PMC11487460 38787275

[CR26] Kapalamula TF, Thapa J, Akapelwa ML, Hayashida K, Gordon SV, Hang’ Ombe BM, Munyeme M, Solo ES, Bwalya P, Nyenje ME, Tamaru A, Suzuki Y, Nakajima C (2021) Development of a loop-mediated isothermal amplification (LAMP) method for specific detection of *Mycobacterium bovis*. PLoS Negl Trop Dis 15:e0008996 10.1371/journal.pntd.0008996 PMC7833227 33493196

[CR27] Kase JA, Pfefer TL (2016) Nucleic acid sample preparation from dairy products and milk. In: Micic M (ed) Sample Preparation Techniques for Soil, Plant, and Animal Samples. Springer Protocols Handbooks. Humana Press, New York, pp 231–244. 10.1007/978-1-4939-3185-9_16

[CR28] Koskinen MT, Holopainen J, Pyorala S, Bredbacka P, Pitkala A, Barkema HW, Bexiga R, Roberson J, Solverod L, Piccinini R, Kelton D, Lehmusto H, Niskala S, Salmikivi L (2009) Analytical specificity and sensitivity of a real-time polymerase chain reaction assay for identification of bovine mastitis pathogens. J Dairy Sci 92:952–959 10.3168/jds.2008-1549 19233788

[CR29] Ku TSN, Al Mohajer M, Newton JA, Wilson MH, Monsees E, Hayden MK, Messacar K, Kisgen JJ, Diekema DJ, Morgan DJ, Sifri CD, Vaughn VM (2023) Improving antimicrobial use through better diagnosis: The relationship between diagnostic stewardship and antimicrobial stewardship. Infect Control Hosp Epidemiol 44:1901–1908 10.1017/ice.2023.156 37665212

[CR30] Lai MY, Zen LPY, Abdul Hamid MH, Jelip J, Mudin RN, Ivan VJS, Francis LNP, Saihidi I, Lau YL (2023) Point-of-Care Diagnosis of Malaria Using a Simple, Purification-Free DNA Extraction Method Coupled with Loop-Mediated Isothermal Amplification-Lateral Flow. Trop Med Infect Dis 8:199 10.3390/tropicalmed8040199 PMC10140920 37104326

[CR31] Land Kevin J, Debrah I, Boeras X-S, Chen AR, Ramsay, Rosanna W, Peeling (2019) REASSURED diagnostics to inform disease control strategies, strengthen health systems and improve patient outcomes. Nat Microbiol 4:46–54 10.1038/s41564-018-0295-3 PMC7097043 30546093

[CR32] Lee S, Khoo VSL, Medriano CAD, Lee T, Park SY, Bae S (2019) Rapid and in-situ detection of fecal indicator bacteria in water using simple DNA extraction and portable loop-mediated isothermal amplification (LAMP) PCR methods. Water Res 160:371–379 10.1016/j.watres.2019.05.049 31163314

[CR33] MacWilliams Maria P, Min K, Liao (2006) Luria broth (LB) and Luria agar (LA) media and their uses protocol. ASM MicrobeLibrary. American Society for Microbiology, 2006: 1–4

[CR34] Moehling TJ, Choi G, Dugan LC, Salit M, Meagher RJ (2021) LAMP Diagnostics at the Point-of-Care: Emerging Trends and Perspectives for the Developer Community. Expert Rev Mol Diagn 21:43–61 10.1080/14737159.2021.1873769 33474990

[CR35] Notomi T, Okayama H, Masubuchi H, Yonekawa T, Watanabe K, Amino N, Hase T (2000) Loop-mediated isothermal amplification of DNA. Nucleic Acids Res 28:E63 10.1093/nar/28.12.e63 PMC102748 10871386

[CR36] Nwe MK, Jangpromma N, Taemaitree L (2024) Evaluation of molecular inhibitors of loop-mediated isothermal amplification (LAMP). Sci Rep 14:5916 10.1038/s41598-024-55241-z PMC10928092 38467647

[CR37] Pascual-Garrigos A, Maruthamuthu MK, Ault A, Davidson JL, Rudakov G, Pillai D, Koziol J, Schoonmaker JP, Johnson T, Verma MS (2021) On-farm colorimetric detection of *Pasteurella multocida, Mannheimia haemolytica*, and *Histophilus somni* in crude bovine nasal samples. Vet Res 52:126 10.1186/s13567-021-00997-9 PMC8487530 34600578

[CR38] Paul R, Ostermann E, Wei Q (2020) Advances in point-of-care nucleic acid extraction technologies for rapid diagnosis of human and plant diseases. Biosens Bioelectron 169:112592 10.1016/j.bios.2020.112592 PMC7476893 32942143

[CR39] Peckler GL, Adcock SJJ (2025) Evaluating somatic cell count, the California mastitis test, and infrared thermography for subclinical mastitis detection in meat ewes. Res Vet Sci 193:105791 10.1016/j.rvsc.2025.105791 40639016

[CR40] Poussard M, Pant SD, Huang J, Scott P, Ghorashi SA (2025) Comparative evaluation of PCR and loop-mediated isothermal amplification (LAMP) assays for detecting *Pasteurella multocida* in poultry. N Z Vet J 73:134–142 10.1080/00480169.2024.2417921 39448061

[CR41] Preine F, Herrera D, Scherpenzeel C, Kalmus P, McCoy F, Smulski S, Rajala-Schultz P, Schmenger A, Moroni P, Kromker V (2022) Different European perspectives on the treatment of clinical mastitis in lactation. Antibiotics (Basel) 11:1107 10.3390/antibiotics11081107 PMC9404852 36009976

[CR42] Ramuada M, Tyasi TL, Gumede L, Chitura T (2024) A practical guide to diagnosing bovine mastitis: a review. Front Anim Sci 5:1504873

[CR43] Ravine D, Suthers G (2012) Quality standards and samples in genetic testing. J Clin Pathol 65:389–393 10.1136/jclinpath-2011-200519 PMC3337464 22259179

[CR44] Riffon R, Sayasith K, Khalil H, Dubreuil P, Drolet M, Lagacé J (2001) Development of a rapid and sensitive test for identification of major pathogens in bovine mastitis by PCR. J Clin Microbiol 39:2584–2589 10.1128/JCM.39.7.2584-2589.2001 PMC88189 11427573

[CR45] Saeed SI, Kamaruzzaman NF, Gahamanyi N, Nguyen TTH, Hossain D, Kahwa I (2024) Correction: Confronting the complexities of antimicrobial management for *Staphylococcus aureus* causing bovine mastitis: an innovative paradigm. Ir Vet J 77:5 10.1186/s13620-024-00266-z PMC10924334 38461351

[CR46] Servellon FJG, Renaud DL, Osorio BJP, Spence KL, DeVries TJ, Serrenho RC (2025) Validation of a rapid on-farm culture system for group classification of clinical mastitis-causing pathogens. JDS Commun 6:562–566 10.3168/jdsc.2024-0738 PMC12848257 41613197

[CR47] Shome BR, Das Mitra S, Bhuvana M, Krithiga N, Velu D, Shome R, Isloor S, Barbuddhe SB, Rahman H (2011) Multiplex PCR assay for species identification of bovine mastitis pathogens. J Appl Microbiol 111:1349–1356 10.1111/j.1365-2672.2011.05169.x 21972842

[CR48] Shwani Abdulkarim B, Zuo A, Alrubaye J, Zhao, Douglas D, Rhoads (2023) A simple, inexpensive alkaline method for bacterial DNA extraction from environmental samples for PCR surveillance and microbiome analyses. Appl Sci 14:141

[CR49] Silva RCD, de Lima SC, Dos Santos Reis WPM, de Magalhaes JJF, Magalhaes RNO, Rathi B, Kohl A, Bezerra MAC, Pena L (2023) Comparison of DNA extraction methods for COVID-19 host genetics studies. PLoS ONE 18:e0287551 10.1371/journal.pone.0287551 PMC10615309 37903126

[CR50] Sowmya N, Thakur MS, Manonmani HK (2012) Rapid and simple DNA extraction method for the detection of enterotoxigenic *Staphylococcus aureus* directly from food samples: comparison of PCR and LAMP methods. J Appl Microbiol 113:106–113 10.1111/j.1365-2672.2012.05315.x 22519925

[CR51] Syring C, Boss R, Reist M, Bodmer M, Hummerjohann J, Gehrig P, Graber HU (2012) Bovine mastitis: the diagnostic properties of a PCR-based assay to monitor the *Staphylococcus aureus* genotype B status of a herd, using bulk tank milk. J Dairy Sci 95:3674–3682 10.3168/jds.2011-4968 22720925

[CR52] Tanner NA, Zhang Y, Evans TC Jr (2015) Visual detection of isothermal nucleic acid amplification using pH-sensitive dyes. Biotechniques 58:59–68 10.2144/000114253 25652028

[CR53] Tomanic D, Kladar N, Kovacevic Z (2025) Antibiotic Residues in Milk as a Consequence of Mastitis Treatment: Balancing Animal Welfare and One Health Risks. Vet Sci 12:1159 10.3390/vetsci12121159 PMC12737421 41472139

[CR54] Wilm J, Svennesen L, Ostergaard Eriksen E, Halasa T, Kromker V (2021) Veterinary Treatment Approach and Antibiotic Usage for Clinical Mastitis in Danish Dairy Herds. Antibiotics (Basel) 10:189 10.3390/antibiotics10020189 PMC7918953 33671911

[CR55] Wong SA, Woodgate RG, Pant SD, Ghorashi SA (2020) Rapid detection of *Bovicola ovis* using colourimetric loop-mediated isothermal amplification (LAMP): a potential tool for the detection of sheep lice infestation on farm. Parasitol Res 119:395–401 10.1007/s00436-019-06552-y 31820169

[CR56] Yamin D, Uskokovic V, Wakil AM, Goni MD, Shamsuddin SH, Mustafa FH, Alfouzan WA, Alissa M, Alshengeti A, Almaghrabi RH, Fares MAA, Garout M, Al Kaabi NA, Alshehri AA, Ali HM, Rabaan AA, Aldubisi FA, Yean CY, Yusof NY (2023) Current and Future Technologies for the Detection of Antibiotic-Resistant Bacteria. Diagnostics (Basel) 13:3246 10.3390/diagnostics13203246 PMC10606640 37892067

[CR57] Yang L, Wang L, Lv M, Sun Y, Cao J (2022) Clinical Validation of DNA Extraction-Free qPCR, Visual LAMP, and Fluorescent LAMP Assays for the Rapid Detection of African Swine Fever Virus. Life (Basel) 12:1067 10.3390/life12071067 PMC9320077 35888155

[CR59] Zou Y, Mason MG, Wang Y, Wee E, Turni C, Blackall PJ, Trau M, Botella JR (2017) Nucleic acid purification from plants, animals and microbes in under 30 seconds. PLoS Biol 15:e2003916 10.1371/journal.pbio.2003916 PMC5697807 29161268

